# Association between dietary inflammatory index and Stroke in the US population: evidence from NHANES 1999–2018

**DOI:** 10.1186/s12889-023-17556-w

**Published:** 2024-01-02

**Authors:** Yukang Mao, Jiayi Weng, Qiyang Xie, Lida Wu, Yanling Xuan, Jun Zhang, Jun Han

**Affiliations:** 1 Department of Cardiology, Suzhou Municipal Hospital, Gusu School, The Affiliated Suzhou Hospital of Nanjing Medical University, Nanjing Medical University, 215008 Suzhou, China; 2 Department of Cardiology, the First Affiliated Hospital of Nanjing Medical University, 210029 Nanjing, China; 3 Department of Cardiology, Nanjing First Hospital, Nanjing Medical University, 210006 Nanjing, China; 4 Nanjing University of Chinese Medicine, 210006 Nanjing, China; 5 Department of Infectious Diseases, Affiliated Wuxi Fifth Hospital of Jiangnan University, The Fifth People’s Hospital of Wuxi, 214065 Wuxi, China

**Keywords:** NHANES, DII, Stroke, Cross-sectional study, LASSO

## Abstract

**Background:**

There is an increasing awareness that diet-related inflammation may have an impact on the stroke. Herein, our goal was to decipher the association of dietary inflammatory index (DII) with stroke in the US general population.

**Methods:**

We collected the cross-sectional data of 44,019 participants of the National Health and Nutrition Examination Survey (NHANES) 1999–2018. The association of DII with stroke was estimated using weighted multivariate logistic regression, with its nonlinearity being examined by restricted cubic spline (RCS) regression. The least absolute shrinkage and selection operator (LASSO) regression was applied for identifying key stroke-related dietary factors, which was then included in the establishment of a risk prediction nomogram model, with the receiver operating characteristic (ROC) curve being built to evaluate its discriminatory power for stroke.

**Results:**

After confounder adjustment, the adjusted odds ratios (ORs) with 95% confidence intervals (CIs) for stroke across higher DII quartiles were 1.19 (0.94–1.54), 1.46 (1.16–1.84), and 1.87 (1.53–2.29) compared to the lowest quartile, respectively. The RCS curve showed a nonlinear and positive association between DII and stroke. The nomogram model based on key dietary factors identified by LASSO regression displayed a considerable predicative value for stroke, with an area under the curve (AUC) of 79.8% (78.2–80.1%).

**Conclusions:**

Our study determined a nonlinear and positive association between DII and stroke in the US general population. Given the intrinsic limitations of cross-sectional study design, it is necessary to conduct more research to ensure the causality of such association.

**Supplementary Information:**

The online version contains supplementary material available at 10.1186/s12889-023-17556-w.

## Introduction

Stroke is a global health problem that bothers hundreds of thousands of people and generates immense costs during post-stroke rehabilitation [1]. Notwithstanding the great advancements in stroke diagnosis and treatment in the past decades [2], the global lifetime risk of stroke among adults aged 25 years or older in 2016 increased by 2.1% compared to 1990 [3]. The prevalence of stroke usually climbs with advancing age [4] ; however, the onset age of stroke tends to be younger in recent years, which is believed to be attributable to some modifiable risk factors (e.g., hypertension, hyperlipidemia, obesity, smoking, and drug addiction) [5]. On top of that, accumulating evidence indicates that systemic inflammation—related to either infection or non-infectious etiologies—has a pivotal role in the pathogenesis and progression of stroke [6]. Elevated circulating levels of inflammatory biomarkers (e.g., interleukin-1β (IL-1β), interleukin-6 (IL-6), tumor necrosis factor-α (TNF-α)) caused by aberrant immune activation is closely involved in stroke occurrence [7], while some medications with well-documented anti-inflammatory capacities—such as statins and canakinumab (also known as a humanized monoclonal antibody against IL-1β)—have been proven to lower the risk of stroke [8, 9], reflecting that chronic low-grade inflammatory status is a key event predisposing to stroke.

It is currently accepted that diet may serve as an essential player in the modulation of systemic inflammation [10]. Unhealthy food patterns—a high-fat, high-calorie Western diet is the best known example—can aggravate chronic low-grade inflammation and thus contribute to the development of cardiovascular diseases (CVDs) through promoting abnormal immune activation [11], whereas a Mediterranean diet that abundantly contains fruits, vegetables, nuts, whole grains, and olive oil has been confirmed to be favorable for lowering systemic vascular inflammation, preserving endothelial function, and sustaining cardiovascular fitness [12]. For the purpose of quantifying the inflammatory capacities of diet more comprehensively and accurately, a literature-derived, population-based dietary inflammatory index (DII) was created by Shivappa et al. in 2014 [13], developed as a powerful reference tool for exploring diet-disease relations, and further confirmed to become higher proportionally with increasing circulating inflammatory biomarkers, including IL-1β, IL-6, TNF-α, and CRP, as well as elevated white blood cell (WBC) counts [14–16]. Hitherto, DII has been widely applied in a great amount of publications to evaluate the roles of diet-induced inflammation in the development of various diseases, such as cancer, obesity, diabetes, and inflammatory bowel disease (IBD) [17–19]. Although there have been several epidemiological studies that explored the association between DII and stroke, the current findings appear to be controversial. Most studies supported a positive association of DII score with stroke [20–23], whereas no significant association was also reported [24, 25]. In this case, our primary goal is to revisit the link between DII and stroke in the general population via using cross-sectional data of the National Health and Nutrition Examination Surveys (NHANES), a nationwide survey recruiting non-institutionalized US residents from the entire country in an unprejudiced manner, conducting laboratory examinations following standardized protocols, and employing sophisticated analytic approaches for data processing and quality control, which may, to a large extent, guarantee the authenticity and reliability of the data.

## Methods

### Study population and ethics

**Fig. 1:**
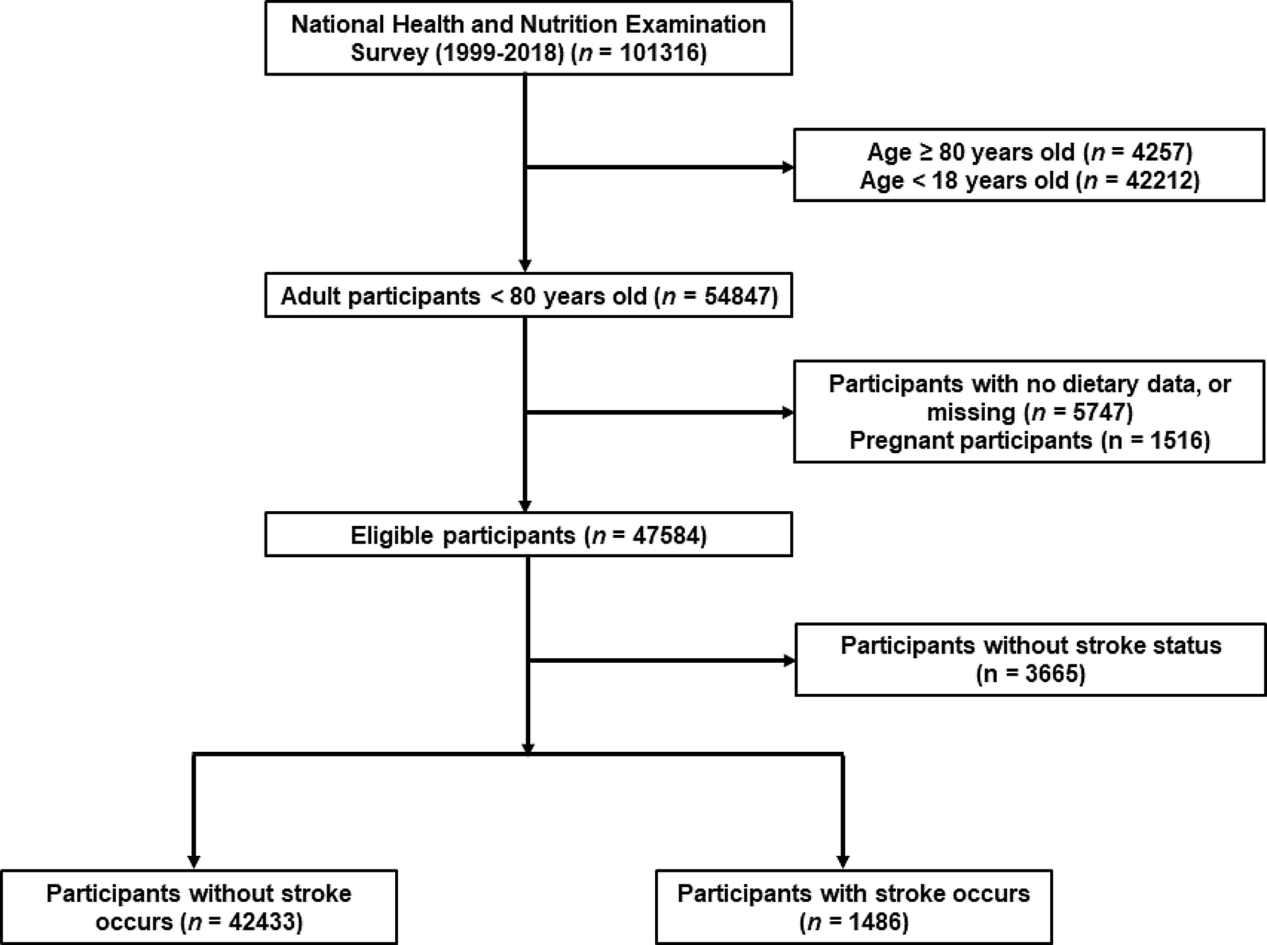
A detailed flow chart of participant recruitment

NHANES is a nationwide campaign launched by National Center for Health Statistics (NCHS) that mainly focuses on the health and nutritional condition of the US civilians at two-year intervals, and whose aim is to obtain a comprehensive knowledge of contemporary disease profiles and to provide references for formulating public health policies [26–28]. All of the NHANES data is accessible to the public and can be downloaded freely through: https://www.cdc.gov/nchs/nhanes/index.htm. In this study, cross-sectional data of 101,316 participants from ten consecutive cycles of the NHANES (1999–2018) were initially included. The exclusion criteria were set as follows: (1) participants aged < 18 or ≥ 80 years (n = 46,469); (2) participants who were pregnant (n = 1,516); (3) participants without relevant information on dietary intake (n = 5,747) and stroke status (n = 3,665). After manual data filtration, we ultimately selected a total of 44,019 participants for subsequent analyses. A detailed flow chart of study participant recruitment was presented in Fig. 1.

### Assessment of dietary information

Dietary intake data regarding the types and amounts of food and drinks consumed during the 24-hour period prior to the interview was recorded in the mobile examination center (MEC), and was used to calculate DII as we previously reported [29, 30]. Concisely, a total of 45 food parameters were taken into account, with each parameter being assigned with a specific DII score depending on their effects on six major inflammatory biomarkers: IL-1β, IL-4, IL-6, IL-10, TNF-α, and CRP [13], and Shivappa and colleagues reported that using no more than 30 food parameters could still be adequate to preserve the predictive value for diet-related inflammation of DII [31]. In the present study, 26 of 45 food parameters were incorporated: energy, protein, carbohydrate, dietary fiber, total fat, saturated fat, monounsaturated fatty acids (MUFAs), polyunsaturated fatty acids (PUFAs), cholesterol, vitamin A, β carotene, vitamin B1, vitamin B2, niacin, vitamin B6, total folate, vitamin B12, vitamin C, vitamin D, vitamin E, magnesium, iron, zinc, selenium, caffeine, and alcohol. To evaluate the inflammatory potentials of one participant’s diet, all food component-specific DII scores were then summed to yield an overall DII, in which a positive score represents pro-inflammatory potential, a negative score represents anti-inflammatory potential, and a zero score represents no significant impact on inflammatory potential, and whose theoretical reference value varies from − 8.87 (diet with maximal anti-inflammatory potential) to + 7.89 (diet with maximal pro-inflammatory potential) [13].

### Assessment of Stroke

Stroke was defined by self-reported previous diagnosis by a physician during face-to-face interview. Anyone who answered “yes” to the following question: “Have you ever been told by a physician or a health professional that you had stroke?” was considered having stroke. To be noted, use of self-reported measures are prone to recall bias, which may have an impact on the interpretation of the data. Besides, despite the lack of relevant information on stroke type in the NHANES database, given the relatively higher prevalence of ischemic stroke among stroke patients as well as its closer relations with chronic low-grade inflammatory status [6, 32], it is likely that the majority of participants with stroke included in this study developed ischemic stroke.

### Covariates

Being based on previous publications and biological considerations, we collected as many covariates with known confounding effects on stroke as possible. Demographic features including age, sex, race/ethnicity, educational level, smoking status, and alcohol consumption were obtained by standardized questionnaires and face-to-face interviews. Physical examination and laboratory tests were performed by experienced medical workers step by step in the MEC.

Race/ethnicity were divided into five categories: non-Hispanic White, non-Hispanic Black, other Hispanic, Mexican American, and other races. The following educational levels were included: below high school, high school, and above high school. Participants who smoked over 100 cigarettes throughout their lifetime were defined as smokers, regardless of whether he/she had quitted smoking at the time of interview [33], and those consuming at least 12 drinks during the year preceding the survey were considered alcohol drinkers [34]. Body mass index (BMI), calculated as weight in kilograms (kg) divided by the square of height in meters (m^2^), is widely used for estimating overweight/obesity status. A BMI score greater than 25 and 30 is recognized as the major diagnostic criteria of overweight and obesity in clinical practice, respectively [35]. Systolic/diastolic blood pressure (SBP/DBP) was measured by experienced clinicians following a standardized procedure, in which three consecutive readings were recorded using mercury sphygmomanometers at half-minute intervals and the mean value of all three readings was designated as one participant’s blood pressure. Fasting blood glucose (FBG), serum concentrations of triglyceride (TG), total cholesterol (TC), low-density lipoprotein cholesterol (LDL-C), high-density lipoprotein cholesterol (HDL-C), hemoglobin (Hb), and glycated hemoglobin (HbA1c), red blood cell (RBC) counts, WBC counts, neutrophil counts, monocyte counts, lymphocyte counts, and platelet counts were determined by standardized laboratory tests. For calculating the estimated glomerular filtration rate (eGFR), NHANES investigators applied a formula developed by the Chronic Kidney Disease Epidemiology Collaboration (CKD-EPI) in which variables including age, sex, race/ethnicity, and serum creatinine (SCr) were incorporated to adapt to different populations [36].

Given hypertension was a critical factor that may predispose to stroke occurrence, it is necessary to rule out its potential confounding effects. Hypertension can be defined based on either self-reported previous diagnosis by a physician or blood pressure measured during physical examination. Participants who met at least one of the criteria listed below was considered having hypertension: (1) Average systolic blood pressure (SBP) ≥ 140 mmHg; (2) Average diastolic blood pressure (DBP) ≥ 90 mmHg; (3) Self-reported diagnosis of hypertension; (4) Current use of anti-hypertensive drugs [37].

In addition, diabetes was another important confounder that may have an impact on stroke. Anyone who provided a previous diagnosis of diabetes by a physician or health professional was defined as patients with diagnosed diabetes, while those without diagnosed diabetes but with a HbA1c level 6.5% (47.5 mmol/mol) or higher, FPG level 126 mg/dL (7.0 mmol/L) or higher, or 2-hour oral glucose tolerance test (OGTT) plasma glucose 200 mg/dL or higher (11.1 mmol/L) tested by laboratory examinations were classified as having undiagnosed diabetes. Participants with diagnosed diabetes or undiagnosed diabetes were both considered diabetic patients [38, 39].

### Statistical analysis

Since NHANES survey employed a series of complex sampling designs, we took into account the sample weights corresponding to different research periods in our analytic methods to yield accurate estimates of health-related statistics [40–42]. Continuous variables were presented in the form of weighted mean and standard deviation (SD), whereas categorical variables were expressed as frequencies and percentages. For the purpose of detecting differences in baseline characteristics between stroke and non-stroke participants, continuous and categorical variables were compared using student’s t-test and chi-square test, respectively. The DII scores were categorized into four quartiles (Q1: DII < 0.23; Q2: 0.23 ≤ DII < 1.76; Q3: 1.76 ≤ DII < 2.95; Q4: DII ≥ 2.95), with the first quartile (Q1) being designated as the reference quartile.

We used a variety of multivariate logistic regression models—non-adjusted model and two adjusted models (Model I and Model II)—to estimate the odds ratios (ORs) and 95% confidence intervals (CIs) for the association of DII with stroke. Adjustments for age, sex, and race/ethnicity was performed in Model I, and Model II was further adjusted for educational level, smoking status, alcohol consumption, BMI, diabetes, and hypertension. We also applied restricted cubic spline (RCS) regression with 3 knots (10th, 50th, and 90th percentiles) to examine the nonlinearity of the association between DII and stroke. Subgroup analyses in terms of age, sex, race/ethnicity, BMI, smoking status, alcohol consumption, diabetes, and hypertension were conducted to examine the presence of significant interactions of these covariates with the association between DII and stroke. To screen the most critical dietary predictors of stroke and eliminate the collinearity among different variables, we applied the least absolute shrinkage and selection operator (LASSO) regression model, in which the coefficients of the variables that make only a negligible contribution to the whole model are shrunk to zero, ensuring the predictive performance of the model [43]. In LASSO model, we used the method of cross-validation for model evaluation and parameter selection where the dataset is divided into 10 subsets, and the model is trained and tested on the 10 subsets multiple times. This helps assess the model’s performance and determine suitable parameter values. During cross-validation, it’s common to plot a curve with respect to lambda values to observe the model’s performance at different lambda settings. “minimum deviance” refers to the lambda value with the smallest bias found during the cross-validation process, which means it provides the best fit to the data. We selected a lambda value slightly larger than the one with minimum deviance, typically by adding one standard deviation to the minimum deviance lambda value. This approach aims to make the model more stable and prevent overfitting, enhancing its generalization to new data. Besides, a risk prediction nomogram model was developed based on several key stroke-related variables, with its discriminatory power for forecasting the risk of stroke being validated by the receiver operating characteristic (ROC) curve. R software version 4.1.6 (http://www.R-project.org, The R Foundation, Vienna, Austria) was used for all statistical analyses, and a two-tailed *P*-value < 0.05 was considered statistically significant.

## Results

### Baseline characteristics of the study participants

In total, 44,019 participants were included in the analysis, with a weighted average age of 45.83 years. The overall prevalence of stroke among all participants was 3.38%, and the weighted median DII score (95% CI) was 1.39 (1.34–1.43). Compared to non-stroke individuals, stroke patients tended to be older, women, non-Hispanic Black, less educated, smokers, non-drinkers, and diabetic patients and had higher levels of systolic blood pressure, triglyceride, total cholesterol, white blood cell counts, monocyte counts, lymphocyte counts, platelet counts, and estimated glomerular filtration rate, as well as lower levels of fasting blood glucose, glycated hemoglobin, low-density lipoprotein cholesterol, high-density lipoprotein cholesterol, and red blood cell counts (all *P* < 0.05). Detailed information about the baseline characteristics of all participants grouped by stroke status was illustrated in Table 1. Based on the finding that stroke group had an exceptionally higher DII score in comparison to non-stroke group (1.99 vs. 1.37, *P* < 0.001), we further investigated the divergence in each dietary component involved in estimating DII between two groups, and found lower intake of nearly all dietary components, with the exception of vitamin E and caffeine, in participants with stroke rather than those without stroke (Table 2). Besides, Supplementary Tables 1 and Supplementary Table 2 presented baseline characteristics of the study participants and several cardiometabolic indexes grouped by DII quartiles, respectively.

**Table 1:** Baseline characteristics of the study participants grouped by stroke status

Variables	Overall (n = 44,019)	Non- Stroke (n = 42,533)	Stroke (n = 1486)	*P* value
Age, years	45.8 [45.5, 46.2]	45.5 [45.1, 45.8]	60.3 [59.4, 61.2]	< 0.001***
Sex-man, *n* (%)	49.3 [47.6, 51.0]	49.4 [48.9, 49.9]	45.3 [42.1, 48.4]	0.01*
Race, *n* (%)				< 0.001***
Non-Hispanic White	68.3 [64.2, 72.3]	68.3 [66.3, 70.3]	66.8 [63.1, 70.4]	
Non-Hispanic Black	11.2 [10.2, 12.1]	11.0 [10.0, 12.1]	16.8 [14.5, 19.1]	
Mexican American	8.2 [7.2, 9.2]	8.3 [7.2, 9.3]	5.3 [4.0, 6.6]	
Other Hispanic	5.7 [4.8, 6.5]	5.7 [4.9, 6.6]	3.6 [2.4, 4.9]	
Other	6.7 [6.1, 7.2]	6.6 [6.1, 7.2]	7.5 [5.5, 9.6]	
Smoking, *n* (%)	22.3 [21.2, 23.3]	22.1 [21.3, 22.8]	29.6 [26.5, 32.7]	< 0.001***
Drinking, *n* (%)	83.4 [80.3, 86.5]	89.31 [88.5, 90.2]	84.3 [81.5, 87.1]	< 0.001***
Education level, *n* (%)				< 0.001***
Below high school	5.4 [5.0, 5.8]	5.3 [4.9, 5.7]	10.3 [8.3, 12.3]	
High school	35.2 [33.5, 37.0]	35.0 [33.8, 36.1]	47.8 [44.5, 51.1]	
Above high school	59.3 [56.9, 61.7]	59.8 [58.4, 61.1]	41.9 [38.4, 45.4]	
SBP, mmHg	121.6 [121.3, 121.9]	121.4 [121.1, 121.7]	130.2 [128.7, 131.7]	< 0.001***
DBP, mmHg	71.7 [71.4, 71.9]	71.7 [71.4, 72.0]	71.0 [70.1, 71.9]	0.13
Diabetes, *n* (%)	12.3 [11.8, 12.9]	11.8 [11.3, 12.2]	35.7 [32.4, 38.9]	< 0.001***
FBG, mmol/L	95.2 [94.7, 95.7]	95.7 [95.2, 96.2]	77.4 [75.7, 79.0]	< 0.001***
HbA1c, %	4.7 [4.7, 4.7]	4.7 [4.7, 4.7]	4.6 [4.6, 4.6]	< 0.001***
eGFR, ml/min/1.73m^2^	7.3 [7.2, 7.3]	7.3 [7.2, 7.3]	7.5 [7.4, 7.7]	< 0.001***
TG, mmol/L	4.30 [4.27, 4.33]	4.29 [4.26, 4.32]	4.59 [4.48, 4.70]	< 0.001***
TC, mmol/L	0.56 [0.56, 0.56]	0.56 [0.56, 0.56]	0.59 [0.58, 0.61]	< 0.001***
LDL-C, mmol/L	2.15 [2.13, 2.16]	2.15 [2.14, 2.17]	2.07 [2.03, 2.12]	0.001**
HDL-C, mmol/L	254.9 [253.5, 256.2]	255.0 [253.7, 256.4]	248.6 [242.4, 254.8]	0.04*
RBC, ×10^9^/L	14.37 [14.33, 14.41]	14.38 [14.34, 14.42]	14.01 [13.89, 14.13]	< 0.001***
WBC, ×10^9^/L	45.8 [45.5, 46.2]	45.4 [45.1, 45.8]	60.3 [59.4, 61.2]	< 0.001***
NE, ×10^9^/L	49.3 [47.6, 51.0]	49.4 [48.9, 49.9]	45.3 [42.1, 48.4]	0.01*
Monocyte, ×10^9^/L	0.56 [0.56, 0.56]	0.56 [0.56, 0.56]	0.59 [0.58, 0.61]	< 0.001***
LY, ×10^9^/L	68.3 [64.2, 72.3]	68.3 [66.3, 70.3]	66.8 [63.1, 70.4]	0.001**
PLT, ×10^6^/L	11.2 [10.2, 12.1]	11.0 [10.0, 12.1]	16.8 [14.5, 19.1]	0.04*
Hemoglobin, g/L	8.2 [7.2, 9.2]	8.3 [7.2, 9.3]	5.3 [4.00, 6.6]	< 0.001***

Continuous variables are presented as weighted mean [95% CI], and categorical variables are presented as unweighted frequencies or percentages [95% CI]. CI, confidence interval; SBP, systolic blood pressure; DBP, diastolic blood pressure; FBG, fasting blood glucose; HbA1c, glycated hemoglobin; eGFR, estimated glomerular filtration rate; TG, triglyceride; TC, total cholesterol; LDL-C, low-density lipoprotein cholesterol; HDL-C, high-density lipoprotein cholesterol; RBC, red blood cell; WBC, white blood cell; NE, neutrophil; LY, lymphocyte; PLT, platelet. * *P* value < 0.05, ** *P* value < 0.01, *** *P* value < 0.001

**Table 2:** Dietary intake of each DII component grouped by stroke status

Variables	Overall (n = 44,019)	Non-stroke (n = 42,533)	Stroke (n = 1,486)	*P* value
DII	1.39 [1.34, 1.43]	1.37 [1.33, 1.41]	1.99 [1.88, 2.11]	< 0.001***
Energy, kcal	2139.1 [2126.2, 2152.1]	2146.6 [2133.8, 2159.4]	1846.1 [1790.4, 1901.8]	< 0.001***
Protein, g	82.9 [82.3, 83.4]	83.2 [82.6, 83.7]	70.8 [68.3, 73.3]	< 0.001***
Carbohydrate, g	257.1 [255.5, 258.8]	257.9 [256.3, 259.5]	226.2 [219.1, 233.4]	< 0.001***
Dietary fiber, g	16.7 [16.5, 16.9]	16.7 [16.5, 17.0]	14.4 [13.8, 15.0]	< 0.001***
Total fat, g	82.0 [81.4, 82.7]	82.3 [81.7, 82.9]	72.6 [69.8, 75.4]	< 0.001***
Total saturated fat, g	26.8 [26.6 27.1]	26.9 [26.7, 27.1]	24.2 [23.2, 25.2]	< 0.001***
MUFA, g	29.7 [29.5, 29.9]	29.8 [29.5, 30.0]	26.1 [25.1, 27.2]	< 0.001***
PUFA, g	18.1 [18.0, 18.3]	18.2 [18.0, 18.4]	15.7 [15.0, 16.4]	< 0.001***
Cholesterol, mg	291.0 [288.3, 293.8]	291.6 [288.8, 294.3]	270.5 [255.5, 285.5]	0.01*
Vitamin A, mcg	637.3 [626.3, 648.4]	638.7 [627.6, 649.8]	582.7 [547.9, 617.6]	0.002**
β carotene, mcg	2238.9 [2162.9, 2314.9]	2249.0 [2172.4, 2325.6]	1848.0 [1640.9, 2055.0]	< 0.001***
Vitamin B1, mg	1.64 [1.63, 1.66]	1.65 [1.63, 1.66]	1.44 [1.39, 1.49]	< 0.001***
Vitamin B2, mg	2.1 [2.15, 2.19]	2.17 [2.15, 2.20]	1.99 [1.91, 2.06]	< 0.001***
Niacin, mg	25.2 [25.1, 25.5]	25.4 [25.2, 25.5]	21.5 [20.7, 22.3]	< 0.001***
Vitamin B6, mg	2.05 [2.03, 2.07]	2.06 [2.04, 2.08]	1.78 [1.70, 1.87]	< 0.001***
Total, folate, mcg	405.9 [401.3, 410.4]	407.3 [402.8, 411.8]	349.7 [335.0, 364.4]	< 0.001***
Vitamin B12, mcg	5.2 [5.1, 5.3]	5.2 [5.2, 5.3]	4.7 [4.4, 5.1]	0.004**
Vitamin C, mg	85.2 [83.5, 86.9]	85.4 [83.7, 87.2]	76.9 [71.7, 82.0]	0.002**
Vitamin D, mcg	4.64 [4.55, 4.73]	4.65 [4.56, 4.74]	4.23 [3.83, 4.64]	0.05*
Vitamin E, mg	66.6 [61.3, 71.8]	66.8 [61.4, 72.2]	56.0 [38.3, 73.8]	0.27
Magnesium, mg	297.7 [294.7, 300.6]	298.7 [295.8, 301.7]	255.3 [247.3, 263.4]	< 0.001***
Iron, mg	15.3 [15.2, 15.4]	15.3 [15.2, 15.5]	13.8 [13.2, 14.4]	< 0.001***
Zinc, mg	11.8 [11.7, 11.9]	11.8 [11.7, 12.0]	10.6 [10.1, 11.0]	< 0.001***
Selenium, mcg	113.1 [112.2, 113.9]	113.5 [112.6, 114.3]	96.0 [92.4, 99.7]	< 0.001***
Caffeine, mg	180.1 [175.6, 184.6]	179.7 [175.3, 184.1]	198.6 [176.0, 221.3]	0.09
Alcohol, g	10.02 [9.58, 10.47]	10.17 [9.71, 10.62]	4.34 [3.30, 5.39]	< 0.001***

Data of dietary intake of DII components are presented as weighted mean [95% CI]. DII, dietary inflammatory index; MUFA, monounsaturated fatty acid; PUFA, polyunsaturated fatty acid. * *P* value < 0.05, ** *P* value < 0.01, *** *P* value < 0.001

### Association of DII with Stroke

**Fig. 2:**
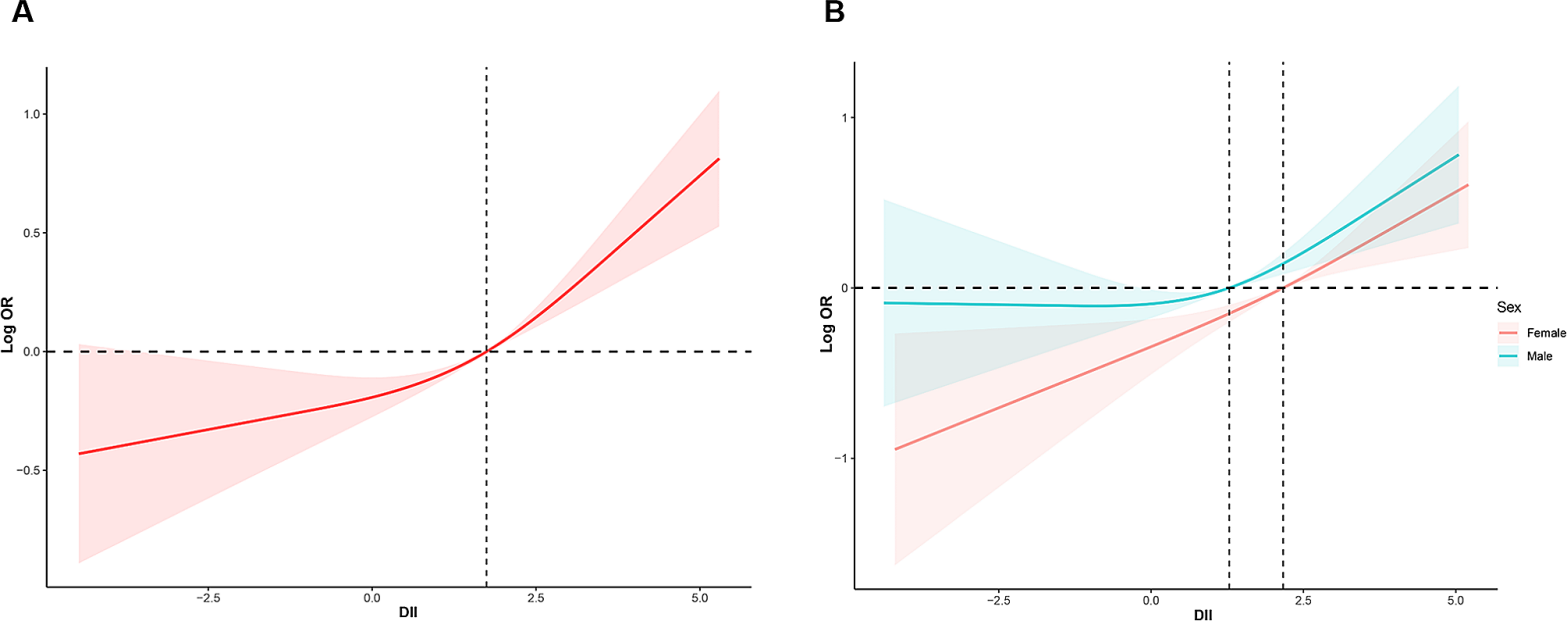
(**A**) The RCS curve of the association between DII and stroke among all the study participants. RCS regression was adjusted for age, sex, race/ethnicity, educational level, smoking status, alcohol consumption, BMI, diabetes, and hypertension. (**B**) The RCS curves of the association between DII and stroke among women (red curve) and men (blue curve), respectively. RCS, restricted cubic spline; DII, dietary inflammatory index; BMI, body mass index; OR, odds ratio

**Fig. 3:**
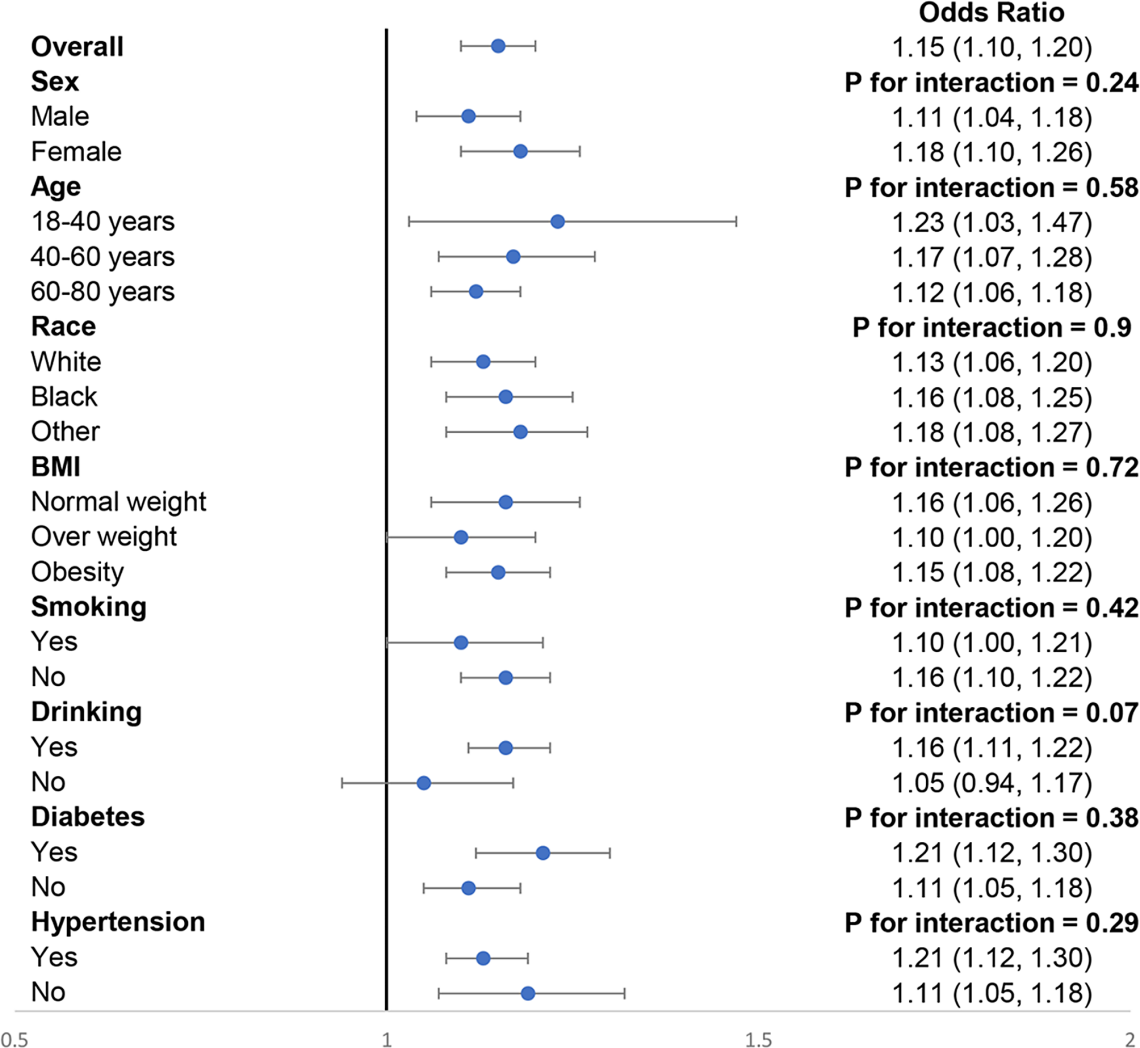
Subgroups analyses for the association between DII and stroke. Analyses were stratified for sex (men and women), age (≤ 40 years, 40–60 years, and ≥ 60 years), race/ethnicity (Black, White, and others), BMI (normal weight, overweight, and obesity), smoking status (smoker and non-smoker), alcohol consumption (drinker and non-drinker), diabetes (yes and no), and hypertension (yes and no). Model II was used in subgroup analysis, which was adjusted for age, sex, race/ethnicity, educational level, smoking status, alcohol consumption, BMI, diabetes, and hypertension. DII, dietary inflammatory index; BMI, body mass index

As shown in Table 3, we performed a sampling-weighted multivariate logistic regression analysis for detecting the association between DII and stroke, and observed that a higher DII was correlated with stroke. After adjustment of potential confounders, the adjusted ORs with 95% CIs for stroke in increasing quartiles of DII were 1.19 (0.94–1.54), 1.46 (1.16–1.84), and 1.87 (1.53–2.29) compared to the lowest quartile, respectively. RCS curve displayed a nonlinear and positive association of DII with stroke (Fig. 2A). We also explored whether a potential gender difference exists in the influence of DII on stroke. In men, the association between DII and stroke remained nonlinear, with the odds of stroke appearing to increase more rapidly when DII exceeded 2, while stroke was found to be linearly correlated with DII in women (Fig. 2B). We also performed stratified analysis to explore whether the association between DII and stroke remained stable across different subgroups. As illustrated in Fig. 3, none of the stratifying variables—including sex (men and women), age (≤ 40 years, 40–60 years, and ≥ 60 years), race/ethnicity (Black, White, and others), BMI (normal weight, overweight, and obesity), smoking status (smoker and non-smoker), alcohol consumption (drinker and non-drinker), diabetes (yes and no), and hypertension (yes and no)—significantly affected the association between DII and stroke (all *P* for interaction > 0.05).

**Table 3:** Weighted logistic regression analysis on the association between DII and stroke

	Non-adjusted model		Model I		Model II	
	**OR [95% CI]**	***P*** **value**	**OR [95% CI]**	***P*** **value**	**OR [95% CI]**	***P*** **value**
DII	1.22 [1.17, 1.27]	< 0.001***	1.22 [1.17, 1.27]	< 0.001***	1.15 [1.10, 1.20]	< 0.001***
Q1	Reference	-	Reference	-	Reference	-
Q2	1.26 [1.01, 1.57]	0.04*	1.26 [1.01,1.58]	0.002**	1.19 [0.94, 1.54]	0.15
Q3	1.69 [1.36, 2.10]	< 0.001***	1.68 [1.34, 2.10]	< 0.001***	1.46 [1.16, 1.84]	0.002**
Q4	2.47 [2.08, 2.94]	< 0.001***	2.43 [2.02, 2.93]	< 0.001***	1.87 [1.53, 2.29]	< 0.001***

Data are presented as OR [95% CI]. Model I was adjusted for age, sex, and race/ethnicity, and Model II was adjusted for age, sex, race/ethnicity, educational level, smoking status, alcohol consumption, BMI, diabetes, and hypertension. OR, odds ratio; CI, confidence interval; DII, dietary inflammation index; Q1, 1st quartile; Q2, 2nd quartile; Q3, 3rd quartile; Q4, 4th quartile. * *P* value < 0.05, ** *P* value < 0.01, *** *P* value < 0.001

### Identification of key stroke-related dietary factors

**Fig. 4:**
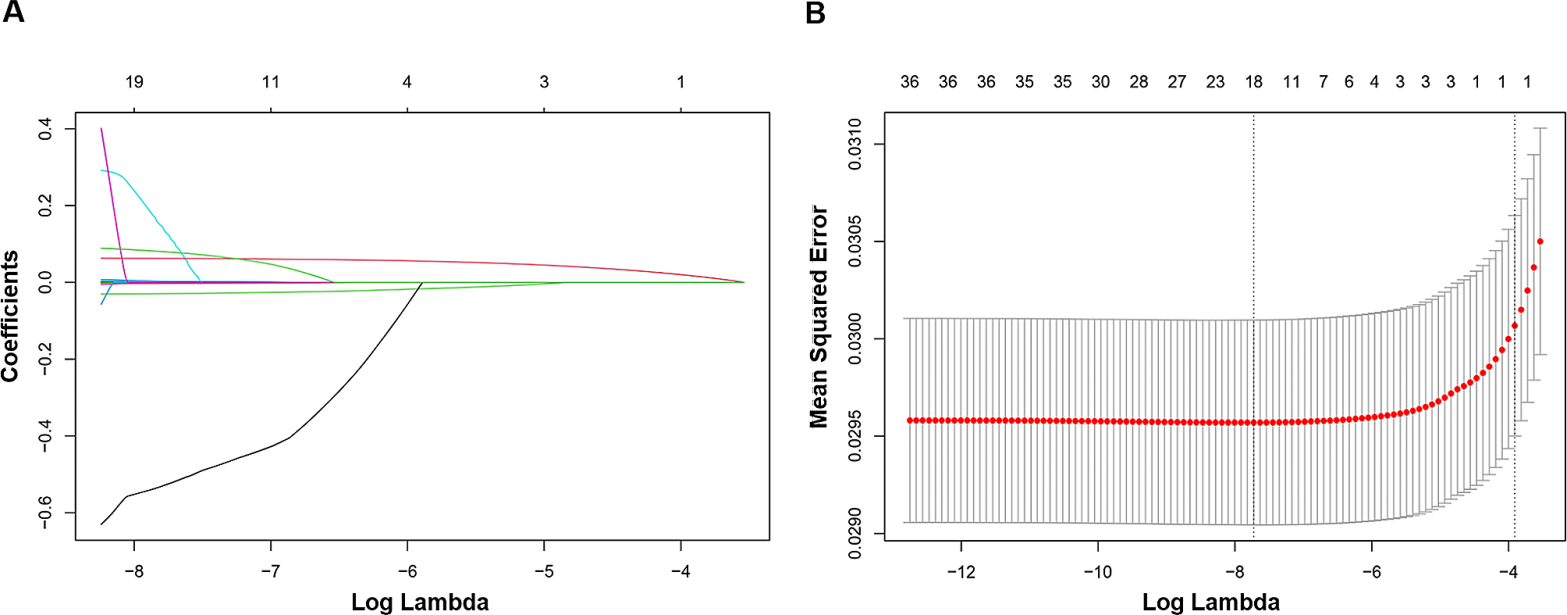
The LASSO penalized regression analysis for identifying key stroke-related dietary factors. (**A**) The coefficient shrinkage process of all 26 dietary components and 3 covariates (sex, age, and race/ethnicity), we represent the changes in coefficients of different features under various levels of shrinkage by drawing lines of different colors. (**B**) A 10-fold cross-validation of the LASSO regression model. LASSO, least absolute shrinkage and selection operator

**Fig. 5:**
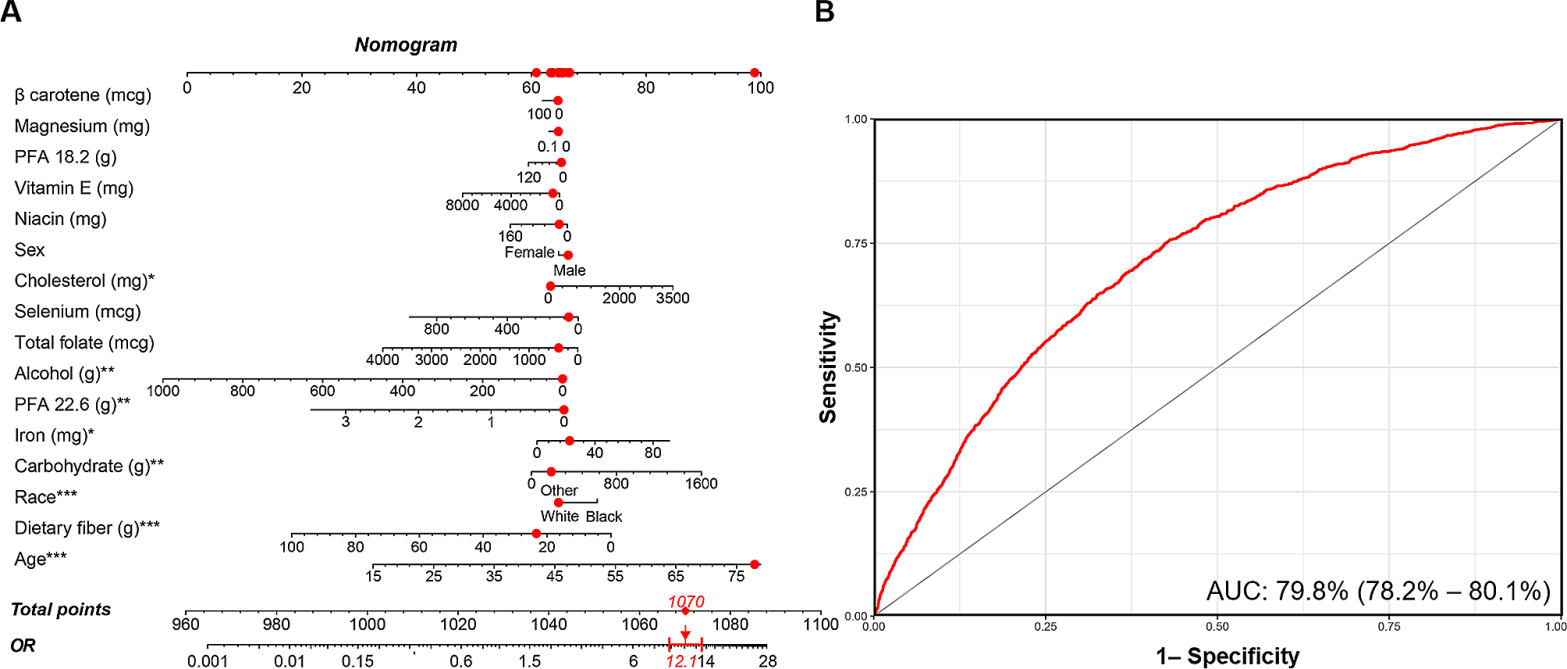
Establishment and validation of a risk prediction model for stroke. (**A**) A nomogram model based on age, race/ethnicity, and 6 key stroke-related dietary factors identified by LASSO regression analysis. (**B**) ROC curve for evaluating the predictive power for stroke of the nomogram model. LASSO, least absolute shrinkage and selection operator; ROC, receiver operating characteristic. * *P* value < 0.05, ** *P* value < 0.01, *** *P* value < 0.001

A risk prediction model based on LASSO penalized regression, in which all 26 dietary components and 3 covariates (sex, age, and race/ethnicity) were incorporated, was created to identify the food parameters that possessed the closest relations with stroke (Fig. 4). In LASSO regression, by adding an L1 regularization term (absolute value penalty term) to the ordinary least squares regression, certain coefficients are shrunk towards zero, thereby achieving the goal of selecting the most important features or variables. This helps prevent overfitting, improves the model’s generalization ability, and performs feature selection, especially when dealing with multiple correlated features, to enhance the model’s interpretability and performance. In LASSO regression, coefficient shrinkage is accomplished by minimizing the loss function along with the L1 regularization term, which encourages some coefficients to be reduced to zero, effectively excluding the corresponding features (Fig. 4A). Carbohydrate, dietary fiber, cholesterol, PUFA (22:6 n-3), iron, and alcohol were selected as the dietary factors most intimately correlated with stroke. To establish a risk prediction nomogram model, a total of 16 variables—including age, sex, race/ethnicity, β carotene, magnesium, PUFA (18:2 n-6), vitamin E, niacin, cholesterol, selenium, total folate, alcohol, PUFA (22:6 n-3), iron, carbohydrate, and dietary fiber—were initially included, while the model was ultimately constructed based on 8 of 16 variables that made statistically significant contributions to the predictive capacity of the model: age, race/ethnicity, carbohydrate, dietary fiber, cholesterol, PUFA (22:6 n-3), iron, and alcohol, with its considerable predictive performance for stroke being validated by ROC curve (AUC = 79.8% (78.2–80.1%)) (Fig. 5).

### Sensitivity analysis

In line with the findings of weighted logistic regression, a positive association of DII with stroke was also determined by sensitivity analysis, which was consistently noted in both non-adjusted and adjusted models. In the fully adjusted model (Model II), the odds of having stroke increased with advancing DII score (Q2: 1.25 (1.05–1.50); Q3: 1.42 (1.20–1.68); Q4: 1.89 (1.60–2.23)), suggesting that participants in higher quartiles of DII were more susceptible to stroke (**Supplementary** Table 3). Overall, sensitivity analysis demonstrated the stability and reliability of the results generated by sampling-weighted logistical regression analysis.

## Discussion

Among the 44,019 NHANES participants included in our study, we observed that individuals with stroke had a pronouncedly higher mean DII score than those without stroke. Based on this phenomenon, we confirmed for the first time a nonlinear and positive association between DII and stroke independently of multiple confounding factors in the US general population. Then, subgroup analyses revealed that this association remains stable across the subgroups divided by several covariates. Among the 26 dietary components chosen for calculating DII in this study, we identified 6 dietary factors that was most closely related to the risk of developing stroke, namely, carbohydrate, dietary fiber, cholesterol, PUFA (22:6 n-3), iron, and alcohol. By combining these key stroke-related dietary factors with age and race/ethnicity, a nomogram model was subsequently built and was validated to exhibit a good performance in forecasting stroke.

It is generally recognized that the occurrence of stroke, ischemic stroke in particular, is largely attributed to a chronic inflammatory state, in which systemic and vascular inflammation induced by infection or non-infectious factors given rise to oxidative stress, endothelial dysfunction, vascular wall injury, platelet activation and aggregation, and eventually intravascular thrombus formation [7]. Dietary intake, as one of the most important modifiable risk factors that affect cardiovascular health, has been demonstrated to exert a double-edged effect in the development of stroke via regulating immune responses and inflammatory reactions, with highly pro-inflammatory diet (e.g., Western diet) on one side and highly anti-inflammatory diet (e.g., Mediterranean diet) on the other [10]. The industrialized Western diet—rich in refined grains, processed meats, animal fats, salt, and food additives and poor in dietary fibers, vitamins, and minerals—is a nutritionally imbalanced dietary pattern that accompanies the advent of modern lifestyle. Several gradients in Western diet, especially cholesterol and saturated/non-saturated fatty acids (FAs), have been well known to stimulate innate immune activation and perpetuate inflammation in a direct manner [44, 45], while these substances can also indirectly shift the immune balance towards pro-inflammatory response through altering the gut microbiota composition and its metabolites [46]. In contrast, Mediterranean diet that highlights adequate intake of whole grains, fruits, vegetables, legumes, nuts, and seafood and advocates for the use of plant oils, especially olive oil, instead of animal oils for cooking represents a healthier dietary pattern and produces considerable cardiovascular benefits. According to a recent meta-analysis that includes 22 randomized controlled trials (RCTs), adoption of a Mediterranean diet may dramatically reduce the serum concentrations of various pro-inflammatory cytokines, including IL-1β, IL-6, IL-8, TNF-α, and CRP, reflecting the capacity of this dietary pattern to suppress systemic inflammation [47]. Another meta-analysis of 20 prospective cohort studies incorporating 682,149 participants and 16,739 stroke cases from different countries and regions suggested that adherence to Mediterranean diet was similarly related to reduced risk of both ischemic stroke (OR 0.86, 95% CI: 0.81–0.91) and hemorrhagic stroke (OR 0.83, 95% CI: 0.74–0.93) [48]. When focusing on assessing the impact of different dietary patterns with varying inflammatory potentials on stroke by DII score, most studies implied that a higher DII score was strongly correlated with stroke, which was completely in line with our results, as well as associated with an increasing rate of mortality and vulnerable carotid plaque in patients with ischemic stroke [49, 50]. In a large population-based prospective study conducted in Korea, having a higher DII score was shown to remarkably increase the risk of developing stroke among men (OR 2.06, 95% CI: 1.07–3.98), which was somehow not observed in women [22]. During nearly the same period, another study incorporating 3,469 postmenopausal women from the Women’s Health Initiative (WHI) revealed that an increase in DII score may solely render women who were overweight more susceptible to stroke (OR 1.32, 95% CI: 1.07–1.64) [23]. Apart from longitudinal studies, similar conclusion is reported by a Kurdish population-based cross-sectional study, in which participants in the highest DII quartile had 1.4 times the odds of developing stroke compared to their counterparts in the lowest quartile (OR 1.4, 95% CI: 1.1–1.8) [21], and is also supported by a RCT demonstrating that stroke risk may constantly increase across with increasing DII quartiles in the fully adjusted logistic regression model (Q2: 1.42 (0.97–2.09); Q3: 1.85 (1.27–2.71); Q4: 1.73 (1.15–2.60)) [20]. Notwithstanding the aforementioned findings, two other studies failed to observe any significant association between DII and stroke [24, 25]. A reasonable explanation for the discrepancies in these findings may be the heterogeneity of study populations, predominantly involving differences in gender, age, country/region, race/ethnicity, and so on, and divergent dietary components selected for calculating DII. Interestingly, apart from affecting stroke itself, diet can also exert substantial effects on post-stroke sequelae. For instance, diets with greater pro-inflammatory potentials were reported to be strongly related to a higher incidence of depressive symptoms and leukoaraiosis in stroke survivors [51, 52], which hampers the rehabilitation course, jeopardizes the quality of life, and shortens life expectancy [53]; however, adherence to the a combination of the Mediterranean diet and another dietary pattern beneficial for improving hypertension, namely the Dietary Approaches to Stop Hypertension (DASH) diet can delay the development of cognitive decline and dementia following stroke [54]. Taken together, since diet-derived inflammation plays a pivotal role in stroke pathogenesis, developing a healthy eating habit is indispensable for the maintenance of cardiovascular fitness, the primary and secondary prevention of stroke, and the promotion of the recovery process after stroke.

The specific dietary components with crucial contributions to the inflammatory potential of a dietary pattern is also noteworthy. According to our analysis, carbohydrate, cholesterol, PUFA (22:6 n-3), dietary fiber, iron, and alcohol constituted key dietary factors for the prediction of stroke. The direct impact of high-carbohydrate diet on stroke remains largely uncertain, with the majority of studies demonstrating no significant association between carbohydrate intake and stroke [55–60] and only two studies reporting a positive [61] or inverse association [62] between the two, respectively. High cholesterol intake, an important hallmark of Western diet, can result in hypercholesterolemia, wherein excessive cholesterol, especially LDL-C, accumulates into macrophages and drives inflammatory response through various mechanisms, which include amplifying Toll-like receptor (TLR) signaling [63], activating NOD-, LRR- and pyrin domain-containing 3 (NLRP3) inflammasome [64], and stimulating the proliferation and maturation of monocytes and neutrophils in the bone marrow and spleen [65], thereby fueling the development of atherosclerotic CVD (ASCVD), including stroke. Cholesterol-lowing therapies using statins or proprotein convertase subtilisin/kexin 9 (PCSK9) inhibitors have been confirmed to mitigate stroke risk proportionally with the degree of reduction in cholesterol levels [8, 66, 67]. PUFAs are organic acids that naturally incorporate two or more double bonds in the aliphatic chain, with the number, configuration, and position of the double bonds being major determinants for naming PUFAs and biologically categorizing these compounds into two families: the n-3 (e.g., docosahexaenoic acid (DHA) (22:6 n-3)) and the n-6 PUFAs (e.g., linoleic acid (LA) (18:2 n-6)) [68]. Since the pivotal roles of PUFAs in multiple biological processes and the lack of PUFA-producing capacity in mammals, consuming food enriched in PUFAs such as plant oil, nuts, and fish thus become a predominant means to acquire PUFAs. Although the effects of PUFAs on stroke remain debatable, a consider amount of studies supported that PUFAs, especially n-3 PUFAs, are intimately linked with diminished risk of stroke [69, 70], suggesting that PUFAs supplementation appears to provide a promising measure for stroke prevention and treatment. In our study, DHA is the only PUFA found to be effective in predicting stroke risk, which may largely be owing to its anti-inflammatory and anti-oxidant properties [71]. Dietary fiber, a class of carbohydrates primarily derived from plant food (e.g., whole grains, fruits, vegetables, nuts), is hard to be digested and absorbed by the gastrointestinal tract and thus cannot serve as an energy source for the human body; however, it plays an unneglectable role in maintaining cardiometabolic homeostasis. Numerous studies have consistently documented an inverse correlation between dietary fiber intake and the risk of stroke [72–75] as well as an inverse association of dietary fiber intake with inflammatory biomarkers [76–78]. Iron is an essential metal element widely participating in multiple biological processes, while current evidence regarding how iron status affects the prevalence of stroke remains poorly understood and conflicting, with both iron deficiency [79] and iron overload [80] being reported to be associated with elevated stroke risk. Thus, more research are necessary to shed light on the underlying mechanisms linking iron status with stroke occurrence. A proper amount of alcoholic beverages, wine in particular, is recommended in a framework of Mediterranean diet due to its anti-inflammatory capacities [81]. Mild to moderate alcohol consumption has been discovered to be inversely correlated with ischemic stroke, while heavy drinking was associated with an increased risk of both ischemic and hemorrhagic stroke [82].

As far as we know, this population-based study revealed for the first time that there exists a nonlinear and positive association between DII and stroke in the US general population, which was exempt from potential bias caused by other confounding factors. On top of that, the application of LASSO regression analysis and the establishment of a nomogram model can facilitate us to extract the dietary factors most closely related to stroke occurrence and render accurate prediction of stroke a reality through combining various key stroke-related factors, thus paving the way for assessing the influence of different dietary patterns on the cardiovascular system and identifying high-risk populations for stroke based on monitoring eating habits in clinical practice.

However, we have to admit that our study has several shortcomings that need to be noted and addressed in further research: (1) Due to the inherent restrictions of cross-sectional design, it remains a tough task to infer causality between DII and stroke, which requires to be further validated in more prospective studies with large-scale cohorts; (2) Although we have attempted to screen as much covariates as possible to control for confounding bias, given stroke is a multifactorial disorder that involves multiple genetic and environmental factors, there may still exist some unknown or unidentified confounders that may also have a role in the pathogenesis of stroke as they were not explicitly documented in NHANES database; (3) Since self-reporting is one of the most effective means to conveniently and rapidly obtain relevant information about dietary intake and how frequently stroke occurs among NHANES participants, whereas such an approach may inevitably lead to recall bias owing to the restrictions of self-reported methods. Thus, caution should be taken during the analysis and interpretation of the data; (4) Two major types of stroke (ischemic or hemorrhagic) as well as their subtypes (large-artery stroke, small-vessel stroke, and cardioembolic stroke for ischemic stroke; intracerebral hemorrhagic stroke and subarachnoid hemorrhagic stroke for hemorrhagic stroke) were not explicitly distinguished in the NHANES database; however, heterogenetic etiologies may serve as key factors responsible for the potential differences in the association between DII and stroke. In other words, how to separately detect the link between diet-related inflammatory status and different subtypes of stroke remains a formidable challenge; (5) The differences in age between the stroke patients and the control group may introduce several limitations in our study. Firstly, age-related factors, such as comorbidities, medication usage, and functional impairments, could potentially confound our findings. We have accounted for age as a covariate in our statistical analyses to mitigate this issue, but residual confounding cannot be entirely ruled out. Secondly, the age disparity may limit the generalizability of our findings. Our study population primarily comprises older individuals, and it may not reflect the stroke outcomes in younger populations. We acknowledge that the results may be more applicable to the older adult demographic and may not be easily extrapolated to younger age groups. Lastly, the age difference may impact the comparability of the two groups, potentially affecting the interpretation of our results. It is essential to recognize that age-related differences in stroke risk factors, pathophysiology, and treatment responses might have influenced the observed outcomes. This inherent bias should be taken into consideration when interpreting the implications of our study; (6) considering ince the nomogram model in this study is constructed based on cross-sectional data, the predictive value of such models may be limited, and the results should be interpreted with caution.

## Conclusion

Taken together, we found a nonlinear and positive association between DII and stroke independently of potential confounding factors, identified several key stroke-related dietary factors, and established a nomogram model based on these factors for predicting stroke in the US general population. Given the inherent limitations of cross-sectional study, further research are indispensable to verify the causality of this association and decipher the underlying mechanisms linking diet-associated inflammation and stroke.

### Electronic supplementary material

Below is the link to the electronic supplementary material.

Supplementary Material 1

Supplementary Material 2

Supplementary Material 3

## Data Availability

All data analyzed in the current study are freely accessible on the NHANES website (https://www.cdc.gov/nchs/nhanes/index.htm).

## Declarations

of conflicting interests.

All authors declare that they have no conflicting interests.

## Abbreviations

ASCVD: Atherosclerotic cardiovascular disease
AUC: Area under the curve
BBB: Blood-brain barrier
BMI: Body mass index
CI: Confidence interval
CKD-EPI: Chronic Kidney Disease Epidemiology Collaboration
CRP: C-reactive protein
CVD: Cardiovascular disease
DASH: Dietary Approaches to Stop Hypertension
DBP: Diastolic blood pressure
DHA: Docosahexaenoic acid
DII: Dietary inflammatory index
eGFR: Estimated glomerular filtration rate
FBG: Fasting blood glucose
Hb: Hemoglobin
HbA1c: Glycated hemoglobin
HDL-C: High-density lipoprotein cholesterol
IBD: Inflammatory bowel disease
IL-1β: Interleukin-1β
IL-6: Interleukin-6
LA: Linoleic acid
LASSO: Least absolute shrinkage and selection operator
LDL-C: Low-density lipoprotein cholesterol
Lp-PLA2: Lipoprotein-associated phospholipase A2
MEC: Mobile examination center
MUFA: Monounsaturated fatty acid
NCHS: National Center for Health Statistics
NHANES: National Health and Nutrition Examination Survey
NLRP3: NOD-, LRR- and pyrin domain-containing 3
2h-OGTT: 2-hour oral glucose tolerance test
OR: Odds ratio
PCSK9: Proprotein convertase subtilisin/kexin 9
PUFA: Polyunsaturated fatty acid
RBC: Red blood cell
RCS: Restricted cubic spline
ROC: Receiver operating characteristic
SBP: Systolic blood pressure
SCr: Serum creatinine
SD: Standard deviation
TC: Total cholesterol
TG: Triglyceride
TNF-α: Tumor necrosis factor-α
TLR: Toll-like receptor
WBC: White blood cell
