# Supplementary material for: Association between dietary inflammatory index and Stroke in the US population: evidence from NHANES 1999–2018
Source: BMC Public Health. 2024 Jan 2;24:50. doi: 10.1186/s12889-023-17556-w (PMC10763382; doi:10.1186/s12889-023-17556-w)
Supplement: Supplementary file 1 — Supplementary Material 1 [file 12889_2023_17556_MOESM1_ESM.docx]

**Supplementary Table 1. Baseline characteristics of the study participants grouped by DII quartiles.**

| Variables | DII-Q1 | DII-Q2 | DII-Q3 | DII-Q4 | *P* value |
| --- | --- | --- | --- | --- | --- |
| Age, years | 46.21 [45.68, 46.74] | 45.91 [45.48, 46.34] | 45.66 [45.19, 46.13] | 45.46 [45.05, 45.87] | 0.08 |
| Sex-male, *n* (%) | 62.37 [61.24, 63.49] | 53.62 [52.43, 54.80] | 43.64 [42.41, 44.88] | 35.21 [34.21, 36.21] | <0.001*** |
| Race, *n* (%) |  |  |  |  | <0.001*** |
| Non-Hispanic White | 71.65 [69.70, 73.61] | 68.25 [66.05, 70.45] | 67.09 [64.83, 69.34] | 65.57 [62.78, 68.36] |  |
| Non-Hispanic Black | 7.76 [6.93, 8.59] | 9.88 [8.78, 10.97] | 12.53 [11.19, 13.87] | 15.24 [13.52, 16.96] |  |
| Mexican American | 8.53 [7.39, 9.67] | 9.01 [7.83, 10.18] | 8.00 [6.84, 9.16] | 7.13 [6.00, 8.27] |  |
| Other Hispanic | 5.07 [4.20, 5.94] | 5.81 [4.81, 6.80] | 6.05 [5.01, 7.08] | 5.87 [4.71, 7.02] |  |
| Other | 6.98 [6.23, 7.73] | 7.06 [6.24, 7.89] | 6.34 [5.66, 7.01] | 6.19 [5.47, 6.91] |  |
| Smoking, *n* (%) | 15.77 [14.77, 16.77] | 20.26 [19.20, 21.32] | 24.31 [23.14, 25.48] | 29.98 [28.70, 31.27] | <0.001*** |
| Drinking, *n* (%) | 91.48 [90.39, 92.57] | 89.75 [88.80, 90.71] | 88.76 [87.69, 89.83] | 86.25 [85.20, 87.31] | <0.001*** |
| Education level, *n* (%) |  |  |  |  | <0.001*** |
| Below high school | 4.23 [3.74, 4.72] | 5.46 [4.92, 6.00] | 5.83 [5.26, 6.40] | 6.31 [5.71, 6.92] |  |
| High school | 27.50 [25.97, 29.03] | 33.23 [31.79, 34.67] | 37.55 [36.00, 39.09] | 44.25 [42.63, 45.86] |  |
| Above high school | 68.27 [66.57, 69.97] | 61.31 [59.73, 62.90] | 56.62 [54.97, 58.27] | 49.44 [47.76, 51.12] |  |
| SBP, mmHg | 120.95 [120.47, 121.42] | 121.69 [121.23, 122.14] | 122.02 [121.58, 122.45] | 121.87 [121.35, 122.40] | 0.002** |
| DBP, mmHg | 72.03 [71.66, 72.39] | 71.63 [71.28, 71.97] | 71.74 [71.37, 72.11] | 71.13 [70.71, 71.54] | <0.001*** |
| Diabetes, *n* (%) | 10.82 [10.12, 11.52] | 12.18 [11.35, 13.01] | 12.52 [11.71, 13.32] | 14.11 [13.24, 14.98] | <0.001*** |
| eGFR, ml/min/1.73m^2^ | 95.09 [94.43, 95.75] | 95.13 [94.53, 95.73] | 95.26 [94.62, 95.90] | 95.47 [94.76, 96.18] | 0.76 |
| RBC, ×10^9^/L | 4.77 [4.76, 4.79] | 4.75 [4.73, 4.77] | 4.72 [4.70, 4.73] | 4.66 [4.65, 4.68] | <0.001*** |
| WBC, ×10^9^/L | 6.94 [6.89, 7.00] | 7.19 [7.12, 7.26] | 7.39 [7.33, 7.46] | 7.55 [7.48, 7.62] | <0.001*** |
| NE, ×10^9^/L | 4.08 [4.04, 4.12] | 4.27 [4.22, 4.31] | 4.38 [4.33, 4.43] | 4.51 [4.45, 4.56] | <0.001*** |
| Monocyte, ×10^9^/L | 0.55 [0.55, 0.56] | 0.56 [0.55, 0.56] | 0.56 [0.56, 0.57] | 0.56 [0.56, 0.57] | 0.04* |
| LY, ×10^9^/L | 2.07 [2.04, 2.09] | 2.12 [2.10, 2.15] | 2.19 [2.17, 2.22] | 2.23 [2.20, 2.26] | <0.001*** |
| PLT, ×10^6^/L | 245.33 [243.56, 247.10] | 252.84 [251.00, 254.67] | 259.85 [258.05, 261.64] | 263.19 [261.06, 265.31] | <0.001*** |
| Hemoglobin, g/L | 14.59 [14.54, 14.64] | 14.46 [14.41, 14.51] | 14.30 [14.25, 14.36] | 14.07 [14.01, 14.13] | <0.001*** |

Continuous variables are presented as weighted mean [95% CI], and categorical variables are presented as unweighted frequencies or percentages [95% CI]. CI, confidence interval; SBP, systolic blood pressure; DBP, diastolic blood pressure; FBG, fasting blood glucose; HbA1c, glycated hemoglobin; eGFR, estimated glomerular filtration rate; TG, triglyceride; TC, total cholesterol; LDL-C, low-density lipoprotein cholesterol; HDL-C, high-density lipoprotein cholesterol; RBC, red blood cell; WBC, white blood cell; NE, neutrophil; LY, lymphocyte; PLT, platelet. * *P* value <0.05, ** *P* value <0.01, *** *P* value <0.001.
