# Supplementary material for: Association between dietary inflammatory index and Stroke in the US population: evidence from NHANES 1999–2018
Source: BMC Public Health. 2024 Jan 2;24:50. doi: 10.1186/s12889-023-17556-w (PMC10763382; doi:10.1186/s12889-023-17556-w)
Supplement: Supplementary file 2 — Supplementary Material 2 [file 12889_2023_17556_MOESM2_ESM.docx]

**Supplementary Table 2. Cardiometabolic indexes grouped by DII quartiles.**

| Variables | DII-Q1 | DII-Q2 | DII-Q3 | DII-Q4 | *P* value |
| --- | --- | --- | --- | --- | --- |
| FBG, mmol/L | 5.79 [5.72,5.85] | 5.89 [5.82,5.95] | 5.81 [5.75,5.87] | 5.86 [5.81,5.92] | 0.04* |
| FBI, pmol/L | 72.11 [69.56,74.67] | 77.76 [74.34,81.19] | 80.38 [77.03,83.74] | 80.78 [78.15,83.41] | < 0.0001*** |
| HOMA-IR | 3.31 [3.15,3.46] | 3.76 [3.54,3.98] | 3.73 [3.55,3.91] | 3.78 [3.62,3.94] | < 0.001*** |
| HbA1c, % | 5.52 [5.49,5.54] | 5.57 [5.54,5.60] | 5.58 [5.55,5.60] | 5.61 [5.59,5.63] | < 0.0001*** |
| TG, mmol/L | 1.47 [1.43,1.51] | 1.53 [1.48,1.58] | 1.55 [1.49,1.61] | 1.45 [1.41,1.48] | 0.003** |
| TC, mmol/L | 5.05 [5.01,5.08] | 5.08 [5.06,5.10] | 5.14 [5.10,5.17] | 5.07 [5.03,5.10] | < 0.001*** |
| HDL-C, mmol/L | 1.38 [1.36,1.39] | 1.37 [1.35,1.38] | 1.37 [1.36,1.39] | 1.35 [1.33,1.36] | 0.002** |
| LDL-C, mmol/L | 2.97 [2.94,3.01] | 3.01 [2.97,3.04] | 3.04 [3.00,3.08] | 3.01 [2.97,3.06] | 0.05 |
| CRP, mg/dl | 0.32 [0.30,0.34] | 0.38 [0.36,0.40] | 0.42 [0.40,0.45] | 0.52 [0.49,0.55] | < 0.0001*** |

Data of cardiometabolic indexes are presented as weighted mean [95% CI]. DII, dietary inflammation index; FBG, fasting blood glucose; FBI, fasting blood insulin; HOMA-IR, homeostasis model assessment to evaluate insulin resistance; HbA1c, glycated hemoglobin; TG, triglyceride; TC, total cholesterol; HDL-C, high-density lipoprotein cholesterol; LDL-C, low-density lipoprotein cholesterol; CRP, C-reactive protein. * *P* value <0.05, ** *P* value <0.01, *** *P* value <0.001.
