# Supplementary material for: Association between dietary inflammatory index and Stroke in the US population: evidence from NHANES 1999–2018
Source: BMC Public Health. 2024 Jan 2;24:50. doi: 10.1186/s12889-023-17556-w (PMC10763382; doi:10.1186/s12889-023-17556-w)
Supplement: Supplementary file 3 — Supplementary Material 3 [file 12889_2023_17556_MOESM3_ESM.docx]

**Supplementary Table 3. Unweighted logistic regression analysis on the association between DII and stroke in sensitivity analysis**

|  | Non-adjusted model | | Model I | | Model II | |
| --- | --- | --- | --- | --- | --- | --- |
|  | **OR [95% CI]** | ***P* value** | **OR [95% CI]** | ***P* value** | **OR [95% CI]** | ***P* value** |
| DII | 1.21[1.17, 1.25] | <0.001*** | 1.19[1.16, 1.23] | <0.001*** | 1.15[1.11, 1.19] | <0.001*** |
| Q1 | Reference | - | Reference | - | Reference | - |
| Q2 | 1.30[1.09, 1.54] | 0.003** | 1.29[1.10, 1.53] | 0.002** | 1.25[1.05, 1.50] | 0.01* |
| Q3 | 1.62[1.38, 1.91] | <0.001*** | 1.58[1.35, 1.86] | <0.001*** | 1.42[1.20, 1.68] | <0.001*** |
| Q4 | 2.37[2.04, 2.77] | <0.001*** | 2.23[1.91, 2.60] | <0.001*** | 1.89[1.60, 2.23] | <0.001*** |

Data are presented as OR [95% CI]. Model I was adjusted for age, sex, and race/ethnicity, and Model II was adjusted for age, sex, race/ethnicity, educational level, smoking status, alcohol consumption, BMI, diabetes, and hypertension. OR, odds ratio; CI, confidence interval; DII, dietary inflammation index; Q1, 1st quartile; Q2, 2nd quartile; Q3, 3rd quartile; Q4, 4th quartile. * *P* value <0.05, ** *P* value <0.01, *** *P* value <0.001
